# Efficacy of Three Vaccine Regimens Against Infectious Hematopoietic Necrosis Virus Transmission Potential in Rainbow Trout

**DOI:** 10.3390/vaccines13080864

**Published:** 2025-08-15

**Authors:** Juliette Doumayrou, Mary G. Frazier, Hannah N. Brown, Andrew R. Wargo

**Affiliations:** Virginia Institute of Marine Science, William & Mary, P.O. Box 1346, Gloucester Point, VA 23062, USA; juliette.doumayrou@gmail.com (J.D.);

**Keywords:** vaccine, virus, transmission, shedding, efficacy, co-infection, salmonid

## Abstract

Background: Vaccination is often a highly effective approach for protecting against clinical disease and mortality caused by viruses. However, vaccine efficacy against viral transmission has rarely been assessed, which can provide vital information on the eradication efficacy and sustainability of vaccines in the field. Methods: Here, we evaluated the host mortality, shedding, and direct fish-to-fish transmission protection efficacy of three vaccine regimens (DNA, inactivated, and attenuated) against infectious hematopoietic necrosis virus (IHNV) in rainbow trout. We quantified protection against single- and mixed-genotype IHNV infections when the vaccines were delivered by intramuscular injection, intraperitoneal injection, and bath immersion, respectively, to reflect field conditions. Results: All three vaccine regimens provided significant protection against fish mortality. The DNA vaccine regimen was qualitatively the most protective and the attenuated vaccine regimen the least. However, these three vaccines provided limited protection against viral shedding. Cumulative shedding over the course of the infection was only slightly reduced compared to unvaccinated fish. There was some indication that the viral genotype fish were exposed to influenced vaccine efficacy, perhaps as a result of genetic similarity to the vaccine strain. Likewise, the DNA vaccine reduced direct transmission in fish cohabitation experiments from 100% to 50%. The inactivated and attenuated vaccine had little impact on IHNV transmission. Conclusions: Collectively, our results suggest that existing IHNV vaccines that increase host survival provide minimal virus transmission protection in rainbow trout, which is likely to limit their long-term efficacy in the field. This work contributes to a growing body of evidence that enhancement of the transmission protection of IHNV and other vaccines will likely bolster disease reduction in the field.

## 1. Introduction

Infectious hematopoietic necrosis virus (IHNV) is a pathogen of high economic and conservation importance [1]. The virus infects wild and farmed salmonid fishes throughout the Pacific Northwest of North America, Europe, and Asia [2]. It can cause severe losses with greater than 90% mortality in some locations, commonly at the juvenile life stages [3,4,5,6]. This has prompted investigations into the development of efficient vaccines against IHN disease, some of which are now available for use in aquaculture [7,8,9,10,11,12,13,14,15,16,17,18]. Current IHNV vaccines can be categorized into three major types: live-attenuated, inactivated, and recombinant. These also broadly represent the primary vaccine types used against pathogens in a variety of systems [19].

Live-attenuated and inactivated (also referred to as killed) vaccines were some of the first types to be investigated for IHNV. They showed several advantages, such as easy production in fish cell cultures, storage stability, and relatively low cost of development [8,14,20]. Almost all existing IHNV vaccines are most effective when delivered by injection [20,21,22]. However, live-attenuated vaccines have shown the most promise for inducing protection when delivered to fish through bath immersion in water [18,23,24,25] or nasal injection [26]. Immersion or incorporation into feed are highly desirable methods of vaccine delivery in aquaculture because of reduced fish stress, lower cost, and higher throughput. These approaches also allow for the vaccination of young fish for which injection can be problematic due to their small size [27]. However, even for live-attenuated IHNV vaccines, immersion and oral delivery typically provide weak and inconsistent disease protection [20,23,28]. In general, the efficacy of inactivated and attenuated IHNV vaccines is low regardless of delivery route [18]. Furthermore, live-attenuated vaccines are often transmissible and come with the risks of releasing live virus vaccination particles into the environment or vaccine-induced disease effects [23,24]. Due to these limitations, additional vaccine strategies for IHNV were pursued.

Recombinant IHNV vaccines were quickly developed as an alternative to live-attenuated or inactivated vaccines. To date, recombinant DNA-based vaccines are the most analyzed and effective vaccines against IHNV [18]. These vaccines have consistently shown a rapid immune response and long-lasting, high-level disease protection after inoculation with just nanograms of DNA [10,11,29]. The most studied of these vaccines contains the sequence for the open reading frame of the surface glycoprotein (G) gene of IHNV [9]. A similar vaccine, APEX-IHN^®^ (Intervet Canada Corp., Kirkland, QC, CA), was commercialized by Novartis in 2005 and subsequently approved for use in Canada and the United States [30]. This represents the only IHNV vaccine and one of two DNA vaccines against any pathogen, which are commercially available in North America to date [31,32]. The APEX-IHN^®^ DNA vaccine has been used extensively in Canadian Atlantic salmon farming, where it has bolstered management of the virus [33,34]. New types of IHNV DNA vaccines are also under development with increasing promise [35,36,37,38,39]. Immunity induced by DNA vaccines is partially established a few days post-injection. This involves early non-specific mechanisms, with durable and highly specific protection developing after approximately 30 days and lasting for at least 2 years post-immunization [29,40,41,42,43]. DNA vaccines have demonstrated strong protection against both intra- and cross-genogroup challenge of circulating virus strains in North America [9,10,11,44,45] and Asia [36]. All of these studies focused primarily on clinical disease prevention (typically host mortality) as the measure of vaccine efficacy. Despite high disease protection efficacy, the DNA vaccine remains relatively expensive to produce compared to other vaccine types. Delivery costs are particularly high given that the most effective protection is achieved through intramuscular injection [10,35], although other routes such as oral delivery are being investigated [28]. A benefit to DNA vaccines is that they are highly stable in storage. There is also no risk of DNA vaccine-induced infections, given that the vaccines do not contain live virus, but rather only a small, non-replicating section of the virus genome [46].

Although the disease protection efficacy of IHNV vaccines has been extensively studied, their efficacy at preventing viral infection, shedding, and transmission has rarely been investigated [18]. One study in Atlantic salmon (*Salmo salar*) demonstrated that the APEX-IHN^®^ DNA vaccine can effectively reduce viral transmission [33]. In contrast, we have shown that a similar DNA vaccine is much less effective at preventing viral shedding than disease in rainbow trout (*Oncorhynchus mykiss*). The vaccines provide only small reductions in the probability and intensity of shedding, particularly at high viral exposure dosages [47]. It is likely that the host species was the driver behind the differences observed between Long (2017) [33] and Jones (2020) [47]. Others have also observed host species impacts on IHNV vaccine efficacy [48], and Atlantic salmon are known to be more resistant to the virus [49]. We know of no studies examining the impact of the live-attenuated or inactivated vaccines on viral shedding and transmission or assessing these three vaccine types simultaneously. Furthermore, the impact of mixed-genotype viral infections on vaccine efficacy has not been evaluated for IHNV. For example, vaccine efficacy is typically tested against just one viral strain, despite IHNV diversity being high in the field [50].

Predictions suggest that vaccinated fish populations could still reach a high rate of virus shed per hour and per farm [51]. This may be particularly problematic when hosts are exposed to multiple virus strains because vaccine efficacy may be diminished [52,53,54]. Ultimately, for vaccination to provide adequate herd immunity and pathogen eradication, viral transmission must be reduced [55]. For IHNV, viral transmission occurs through horizontal transfer of virus between fish through shedding into the water; thus, quantification of shedding provides a relevant proxy for transmission [47,56,57,58,59,60]. Vaccines that reduce disease but do not protect against transmission can also have long-term consequences, such as selecting for the evolution of increased virulence [61,62,63] or vaccine resistance [64]. There is thus a critical need for a better understanding of how IHNV vaccines impact viral transmission. Herein, the fish survival, shedding, and direct transmission protection efficacy of three IHNV vaccine types: DNA, inactivated, and attenuated, were investigated in rainbow trout (*Oncorhynchus mykiss*). Protection efficacy against two IHNV genotypes in single and co-infection was also assessed. The vaccines were delivered by intramuscular injection, intraperitoneal injection, and immersion, respectively, to evaluate efficacy under known historical and preferred field usage [1,14,23,27,33]. Each vaccine type–delivery route combination was referred to as a regimen, to reflect these field trends.

## 2. Methods

### 2.1. Vaccines

Three vaccine types, DNA, inactivated, and attenuated, were characterized for their efficacy at reducing fish mortality and viral shedding after IHNV infection. These vaccines were chosen to represent the three major vaccine types most commonly utilized to manage IHNV [18,32,65]. The DNA vaccine was created using a Giga plasmid prep (Qiagen) of bacteria transfected with the plasmid pWg (provided by Gael Kurath, USGS, USA), which contains the glycoprotein (G) gene of IHNV genotype WRAC (Genbank accession number NC_001652) as previously described [9,10,47,56]. A sham DNA vaccine control (pLuc) was created by inserting the luciferase gene into the same vector as the pWg vaccine, instead of the viral G-gene, and then propagated as for the DNA vaccine. The inactivated vaccine was created through treatment of a high-titer (1.52 × 10^8^ plaque-forming units (PFU) per mL) virus stock of genotype WRAC with 2.7 mM β-Propiolactone (BPL, Sigma-Aldrich) at 22 °C for 72 h, as described in Anderson et al. (2008) [14]. BPL was then neutralized by the addition of 1 M sodium thiosulphate to a final concentration of 20 mM. The live-attenuated vaccine was provided by Clear Springs Foods Inc. (Buhl, ID, USA) and was created through passage of virus genotype WRAC through cells. The inactivated and attenuated vaccines were titered for live virus and infectivity by plaque assays on *Epithelioma papulosum cyprinid* (EPC) fish cells [66] and found to have titers of 0 (non-infectious) and 5.08 × 10^8^ PFU (infectious) of virus mL^−1^, respectively. All vaccines were stored at −80 °C until use.

### 2.2. Viruses

The viruses used to infect fish in these experiments were IHNV isolates LR80 (genotype mG007M; GenBank accession no. L40878 [67]) and MER95 (genotype mG111M [68]). Herein, we refer to these as viral genotypes to signify that they were genetically distinct from each other. Sequence alignment (EMBOSS Needle) of available full genomes (obtained from GenBank or USGS Western Fisheries Research Center) revealed 98.2% identity (10935/11134 nucleotides) between LR80 and WRAC and 97.1% identity (10813/11134 nucleotides) between MER95 and WRAC. Isolates WRAC, MER95, and LR80 fall into genogroups MA, MD, and MH, respectively, based on their mid-G sequences [69]. As such, challenge isolates MER95 and LR80 are heterologous to the vaccine strain WRAC, with MER95 being slightly more disparate. Virus stocks were prepared by propagation of the virus on EPC cells, then stored at −80 °C. The stocks were tittered on EPC cells, as previously described [70], and found to be 2.22 × 10^9^ and 9.32 × 10^9^ PFU mL^−1^ for LR80 and MER95, respectively.

### 2.3. Fish

A specific-pathogen-free aquaculture rainbow trout line (*Oncorhynchus mykiss*) was used in these experiments, provided by Clear Springs Foods Inc. A total of 1830 fish were maintained in specific pathogen-free, UV-irradiated freshwater at 15 °C, under flow-through conditions. Fish weighed approximately 0.90 g (±SE 0.07) and 1.02 g (±SE 0.06) for the disease and shedding protection assay experiments described below, respectively. Fish were taken off feed 48 h prior to exposure to the virus, and then fed every other day afterwards, at 1–2% of their body weight with a 0.6–0.85 mm fish diet (Zeigler).

### 2.4. Vaccination

For DNA vaccination, fish were anesthetized by immersion in a 100 mg·L^−1^ tricaine methane sulfonate solution (MS-222, Pentair Aquatic Eco) buffered with 300 mg·L^−1^ sodium bicarbonate (pure baking soda, Arm & Hammer). The fish were then injected intramuscularly (IM) with 50 ng of DNA vaccine in 25 μL phosphate-buffered saline (PBS) using a 30G ½ inch sterile needle on a repeat pipettor. A sham DNA vaccine group was IM injected with 50 ng of luciferase plasmid in 25 μL PBS. For inactivated vaccination, the fish were anesthetized as above and injected intraperitoneally (IP) with the equivalent of 3.8 × 10^5^ PFU mL^−1^ of inactivated virus in 25 μL of media MEM-10 [56] using a 30G ½ inch sterile needle. A sham-inactivated vaccine group was IP injected with 25 μL MEM-10 supplemented with final concentrations of 2.7 mM BPL and 20 mM sodium thiosulphate. For live-attenuated vaccination, fish were immersed in 1 × 10^6^ PFU mL^−1^ of the vaccine in 1 L of static water for 1 h with supplemental aeration, in a 5-gallon bucket. A sham attenuated vaccine group was immersed in 1 L of water containing 5 mL of MEM 10, as described for the attenuated vaccine treatment. Both the inactivated and attenuated vaccines were generated from undiluted high-titer viral stocks to maximize the vaccine dosages delivered. Each vaccination group contained 305 total fish (Table S1). Fish were maintained for 38 (disease protection assays) or 34 (shedding and transmission protection assays) days to allow specific immunity to develop [29] before exposure to the virus. Throughout, sham-vaccinated fish are referred to as unvaccinated treatments for the respective vaccine types.

### 2.5. Disease Protection Assays

To examine how well the three vaccine types protected against IHN disease, vaccinated and unvaccinated fish were exposed to the virus, and mortality was quantified, as previously described [71]. Briefly, triplicate groups of 20 fish per vaccine and virus treatment were placed in 6 L tanks with supplemental aeration containing 995 mL of pathogen-free 15 °C water, in a randomized design. Fish from each vaccine treatment were exposed to single infections of IHNV genotypes LR80 and MER95 alone at a dose of 2 × 10^5^ PFU·mL^−1^, or a mixed infection of 2 × 10^5^ PFU mL^−1^ of each genotype at a 1:1 ratio (treatment named ‘1:1’; 4 × 10^5^ PFU·mL^−1^ total virus) [72]. A virus-free control group for each vaccine treatment, exposed to MEM-10, was also included (see Table S1 for full sample numbers). After a 1-h static water exposure period, water flow was resumed at a rate of 100 mL min^−1^, and fish were maintained for 35 days. Mortalities in each tank were recorded and dead fish removed daily for 35 days post-exposure to the virus. At the end of the experiment, all surviving fish were euthanized with 0.27 mg mL^−1^ Tricaine-S (MS222; Western Chemical, Inc., Ferndale, WA, USA) buffered with 0.09 mg mL^−1^ sodium bicarbonate, and then counted.

### 2.6. Shedding Protection Assays

To quantify how well the three vaccines protected against IHNV shedding, a second experiment was run. A different group of the same lot of fish was vaccinated three weeks after the start of the disease protection experiment. The fish were then exposed to the virus, and water samples from tanks housing individual fish were collected as previously described [47,59,60]. Briefly, vaccinated and unvaccinated fish were exposed to single or mixed infections of virus isolates LR80 and MER95, after vaccination, as for the disease protection assays. After the 1-h static exposure to the virus, the fish were transferred to a new tank and held for 1 h in flowing water (1500 mL·min^−1^), previously demonstrated to remove all residual exposure virus [47]. Afterwards, each individual fish was randomly separated into a 0.8 L tank in a tower rack system (Aquaneering). This allowed for tracking of shedding kinetics at an individual fish level. The virus treatments contained 20 replicate fish, and a mock control group of 5 fish exposed to MEM-10 was included for each vaccine treatment (Table S1). The initial immersion time of the naïve fish with the virus was day 0 in this experiment.

To quantify viral shedding, an 800 μL water sample was collected at day 0 from each individual tank, and water flow was then turned off for 22 h. The water was then sampled again from each tank at day 1 (after the 22 h static period), and the water flow was turned to 150 mL·min^−1^ for a 2-h wash to remove all virus; after which the flow was again turned off for 22 h. This process was repeated for days 2 to 7. After sampling on day 7, tanks were flushed for 2 h, and then the flow was set to a fast drip (approximately 40 mL min^−1^) for 24 h. At day 9, the tanks were flushed again for 2 h, the flow was turned off for 22 h, and then sampled on day 10. As such, water samples provided a daily cumulative amount of virus shed over 22 h. Water samples were stored at −80 °C for further processing. Because prior studies indicate that shedding is a relevant proxy for IHNV transmission [56,73,74], we sometimes refer to shedding as transmission potential throughout. Mortalities were recorded daily for 30 days. Dead fish were kept in their tanks and continued to be sampled until the end of the experiment. Virus detected in dead fish was included in all figures and analyses.

### 2.7. Transmission Protection Assays

To evaluate the efficacy of the three vaccine regimens at protecting against IHNV transmission, cohabitation assays were conducted as previously described [56]. Briefly, groups of 108 rainbow trout (same lot as experiments above, 4 months older) per treatment underwent one of the 6 vaccine regimens (DNA, inactivated, attenuated; either unvaccinated and vaccinated) as described above. After vaccination, the fish (weight = 1.00 ± 0.05 g/fish) were divided in half to create populations of donor and recipient fish (54 fish each per vaccine regimen). Donor fish were infected with a controlled dosage of virus from cell culture stocks, and recipient naïve fish were added to the donor fish tanks to assess transmission.

To infect donor fish, they were exposed to 1 × 10^6^ PFU·mL^−1^ of IHNV genotype LR80 (MER95 was not used for transmission assays) by bath immersion for 3 h, followed by a 1-h wash, as described above. Then, the donor fish from each vaccine regimen were divided into 18, 0.8 L tanks, with 3 donor fish per tank, and held in static water for 24 h. An 800 µL water sample was then taken from each donor fish tank to confirm the average shedding (and thus infection) status of the 3 donor fish in each tank. Afterwards, the unexposed recipient fish from each vaccine regimen were divided into 18 mesh bags (only 17 recipient replicates for the attenuated and inactivated vaccine treatments), with 3 fish per bag. The bags containing the recipient fish were placed in the tanks with the donor fish (same vaccine regimen as donors but not exposed to virus) and held in static water for 24 h. The cohabitation thus occurred 24–48 h after infection of the donor fish, to capture the peak window of shedding [59,60]. The mesh bags allowed for water flow (as well as movement and respiration of fish), and thus transfer of virus, but not direct contact between donor and recipient fish. After the cohabitation, the 3-recipient fish in each mesh bag were then removed from the donor tank and released into a single new 0.8 L tank (18 total tanks per vaccine regimen) with clean water. The tanks were then washed under water flow (300 mL/min) to remove any virus. The recipient fish were held for an additional 48 h in static water, and an 800 µL water sample was taken from each tank to confirm the average viral infection and shedding status of the 3 recipient fish in the tank. All water samples were stored at −80 °C until further processing via quantitative PCR.

### 2.8. Viral Quantification in Water

Total nucleic acids were extracted from 210 μL of each water sample with a Tecan Freedom EVO^®^ 100 liquid handler (Männedorf, Switzerland) using the Cador Pathogen 96 Qiacube HT Kit (Qiagen) and were eluted with 100 μL of elution buffer, according to Doumayrou et al. (2019) [56]. All extracted nucleic acids were stored at −80 °C until further processing. For virus quantification, RNA samples were converted to cDNA as described in Wargo et al. (2010) [72]. The cDNA was stored at −80 °C until genotype-specific quantitative PCR (qPCR) to distinguish LR80 and MER95, targeting the encoding region of the glycoprotein (G-gene) of the virus.

The final qPCR reaction utilized forward primers LR80v2 (5′-GAC TTC TGT GGG AAA CAG TGG ATA C-3′) or MER95v2 (5′-CAT TCT GTG GGA AAC AGT GGA TAA-3′), reverse primers LR80v2 (5′-TCC CCT CAT CCC CAC AGT C-3′) or MER95v2 (5′-TTC CCC TCA TCC CCA CAG TA-3′), and MGB TaqMan probes LR80v2 (5′-MGB-VIC-AAA CTC TCT ACA AAA GAA GCA G-NFQ-3′) or MER9v2 (5′-MBG-6′FAM-CTG GAG CAG AAA CCC-NFQ-3′; Applied Biosystems). Each quantitative PCR was performed in a 12 μL reaction containing 1× TaqMan™ Universal Master Mix II, no UNG (Thermo Fisher Scientific, Waltham, MA, USA), 900 nM respective forward and reverse primers, and 200 nM probe. Each sample was run separately for each assay (LR80v2, MER95v2), without multiplexing. Viral quantification was performed in 384-well optical plates using a QuantStudio6 Flex real-time thermal cycler (Applied Biosystems) with the specific program for each assay (15 min at 95 °C, 40 cycles for 15 sec at 95 °C and 1 min annealing at 61 °C for LR80 or annealing at 58.5 °C for MER95).

RNA transcripts pLR80G and pMER95G, of the G-gene of genotypes LR80 and MER95, were used to generate an 8-step standard curve for each assay [75]. This allowed for the quantification of assay amplification efficiency as well as the amount of virus in the water samples. The qPCR quantified the number of viral RNA copies, presented here as virus copies, per mL of water. For the MER95v2 assay, we had a linear response between 10 and 1 × 10^7^ RNA copies per reaction, with 87.4% ± 0.78% SE efficiency and no detection of possible non-targets (WRAC and LR80) at 1 × 10^7^ copies per well. For the LR80v2 assay, the linear response was between 100 and 1 × 10^7^ RNA copies per reaction, with 87.3% ± 0.61% SE efficiency and no detection of non-targets at 1 × 10^7^ MER95 copies per well and 1 × 10^5^ WRAC copies per well.

### 2.9. Statistical Analysis

Statistical analyses were carried out with R, version 3.3.3 [76], in RStudio Version 2023.12.369 (Posit team, 2023). Multiple model parameterizations were considered that included all combinations of main effects and interactions between covariates for each analysis. The simplest model with the highest explanatory power, which successfully converged, was chosen under the assumption of parsimony. To obtain the best-fit model, different model fits were compared using a likelihood ratio test and AIC values, with differences in AIC value greater than 2 considered significant. For models that did not significantly differ, the one with the fewest parameters was chosen. Models selected are presented in the supplemental materials, and model inferences are presented in the main body text. Data for each vaccine was analyzed separately, because the unvaccinated treatment as well as the delivery methods differed for each vaccine type, so direct quantitative comparisons were not made. The goal was to compare the performance of each vaccine against its respective unvaccinated control, and only make qualitative comparisons across vaccine types. We also analyzed the data for each viral genotype separately, because the efficiencies of qPCR assays for the genotypes were not equal. Again, only qualitative comparisons were made across genotypes. For these analyses, the baselines were set to unvaccinated and single-genotype exposure treatments. The exception to this was for the survival data, where all viral genotypes were analyzed together, and baselines were set to unvaccinated and co-infection treatments. A few fish were removed before the end of the experiment due to severe pathology or escape, and were dropped from the statistical analyses (censored), from the time of removal onward. When best-fit models indicated interaction terms, plots of fitted values with 95% confidence intervals were used to resolve differences between factor levels. Main effects were only considered if the best-fit model did not include the factor in interaction terms. Model results are typically shown as test statistic values with factor and residual degrees of freedom given as a subscript separated by a comma, followed by the *p*-value, for the coefficient of the parameter of interest.

### 2.10. Mortality

Survivorship analysis was conducted on the disease protection assay experiment using Cox proportional hazards regression with the ‘coxme’ function from the package ‘coxme’ in R [77,78]. The response variable was day of death, with censoring of live fish on the last day of the experiment and fish removal from the tank during the experiment. Explanatory factors included vaccine (vaccinated or unvaccinated) and virus (LR80, MER95, or co-infection). Tank was included as a random effect for triplicate tanks within each treatment to account for intra-tank effects on survival. The proportional hazards assumption of the model was validated using the cox.zph function. The relative percent survival (RPS) of each vaccine was estimated at the end of the virulence challenge to qualitative estimate their respective protection efficacy against IHN disease according to [79]: RPS = (1 − (cumulative mortality in VAC fish/cumulative mortality in unvaccinated fish)) × 100%.

### 2.11. Number of Fish Shedding Virus

The number of fish shedding IHNV over time was analyzed on the shedding protection assay experiment with a binomial generalized linear mixed model using the function ‘glmer’ in the R package ‘lme4’ [80]. Explanatory fixed factors included vaccine (vaccinated or unvaccinated), virus (LR80, MER95, or co-infection), and day (continuous). The impact of the inclusion of fish and day as random effects on the model fit was investigated to account for temporal repeated measures in the dataset. The response variable of these analyses was fish infection status for each vaccine type (positive or negative). If models did not converge, a ‘bobyqa’ optimizer was utilized. If similar parameter estimates were found with both approaches, the results from models without optimizers are shown. If neither model converged, the model was discarded. Over-dispersion was investigated with the function ‘binnedplot’ in the R package ‘arm’ and the function ‘dispersion_glmer’ in the R package ‘blmeco’, but was not found to be a significant issue for any models. None of the fish shed at day 0. Thus, the shedding status of all fish (yes or no) was analyzed from day 1 forward. Our goal was to compare how the probability of fish shedding changed from the start of shedding forward.

### 2.12. Shedding Intensity of Virus

The intensity of virus shed over time was analyzed on the shedding protection assay experiment data using a generalized linear mixed model with the function ‘lme’ in the R package ‘nlme’ [81]. The explanatory fixed and random factors in this model were the same as for the number of fish shedding analyses. The response variable was shed Log_10_(viral RNA copies/mL H_2_O +1). Individual fish were excluded from the analysis at time points where no virus shedding was detected from that fish, because the goal was to compare the rate of shedding in only those fish that shed. Days 0–1 were excluded because they lacked sufficient replicate data points due to very little viral shedding. Thus, the analysis compared the intensity of viral shedding from the start of shedding forward. The assumptions of the models were validated using the Bartlett test and normal probability (Q-Q) plots. The varPower weights option was included to control for heteroscedasticity and improve model fit.

### 2.13. Cumulative Virus Load Shed

To compare cumulative virus shedding data from the shedding protection assay experiment, the total amount of each IHNV isolate shed between days 0 and 10 was summed for all fish. A multi-factor ANOVA was then conducted. For each vaccine dataset, the explanatory fixed factors included vaccine (vaccinated or unvaccinated) and virus (LR80, MER95, or co-infection). The response variable was the total amount of virus shed, log10 transformed to adhere to the assumptions of variance homogeneity and normality, which were validated using Levene’s test and the normal probability plot of residuals. Explanatory fixed factors were the same as for the number of fish shedding analyses, with the exclusion of day.

### 2.14. Transmission Data

The efficacy of the vaccine regimens at preventing transmission was evaluated by analyzing the number of fish becoming infected and the amount of virus shed in the cohabitation studies. As above, the analyses were conducted separately for each vaccine regimen. To analyze the number of fish infected, A chi-square test was used to compare the number of replicate recipient cohabitation tanks positive for viral shedding (i.e., number of successful independent transmission events) in vaccinated versus unvaccinated fish. General linear models were also explored for this analysis, but failed to converge due to many treatment groups having 100% of recipient fish infected. To compare viral shedding amounts, mixed effects linear model ‘lme’ analysis was conducted, with the response variable log10(viral RNA copies/mL H_2_O +1) and predictor variables fish type (donor or recipient) and vaccine status (unvaccinated or vaccinated), as well as the random term cohab number (donor–recipient pair) to control for tank effects. Tanks with no detectable shedding were included in this analysis (set to 0), because they were few in number and their inclusion did not violate the normality assumption of residuals (evaluated as for shedding intensity analysis).

## 3. Results

### 3.1. Fish Survival

**Figure 1:**
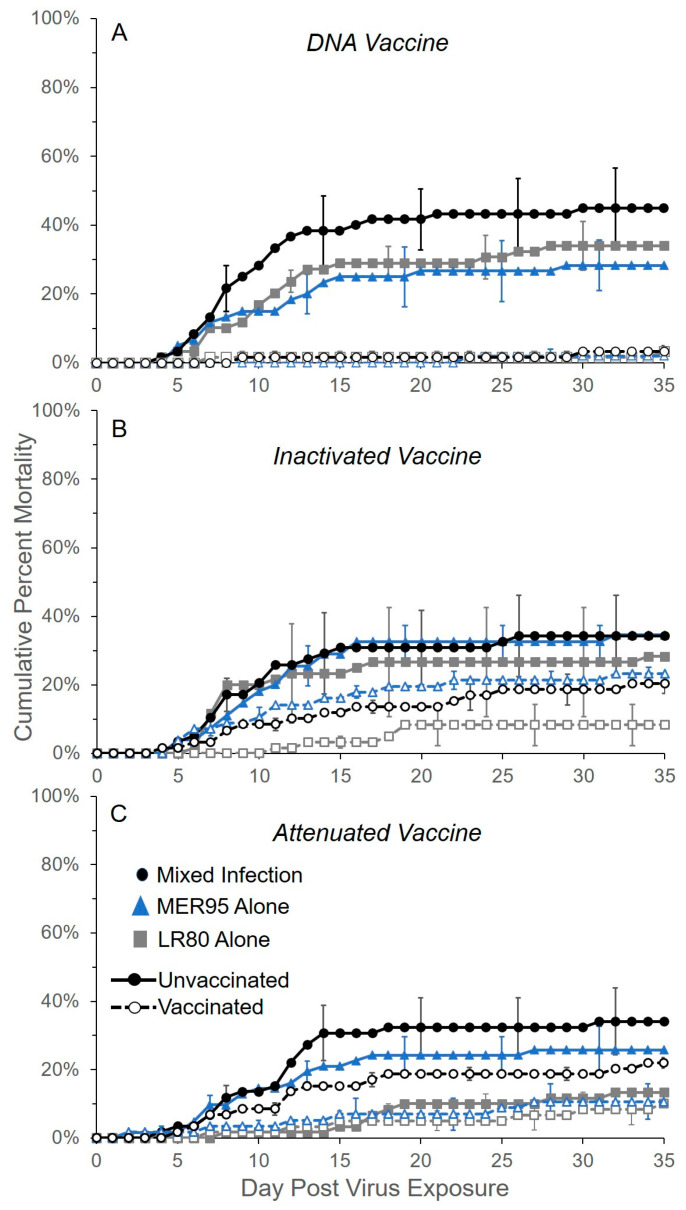
Effect of three vaccine regimens on the survival of fish infected with two IHNV genotypes in single and co-infection. Lines represent cumulative percent mortality (±1 mean standard error) averaged across triplicate groups of 20 fish exposed to IHNV genotype MER95 alone (blue triangle), LR80 alone (gray square), or a mixed infection of the two genotypes at a 1:1 ratio (black circles). Dashed lines (and open symbols) represent fish vaccinated with DNA (**A**), inactivated (**B**), or attenuated (**C**) vaccine regimens, and the solid lines (and closed symbols) represent corresponding unvaccinated control treatments. Legend in panel (**C**) applies to all panels. No fish mortality was observed in any of the mock no virus exposure control groups (21 tanks and 420 fish total across all treatments), with the exception of 1 fish in the DNA unvaccinated treatment, which was found dead on day 34 of the experiment (data not shown). Data is from the disease protection assay experiment (see Methods). SE bars are staggered between lines for readability.

**Figure 2:**
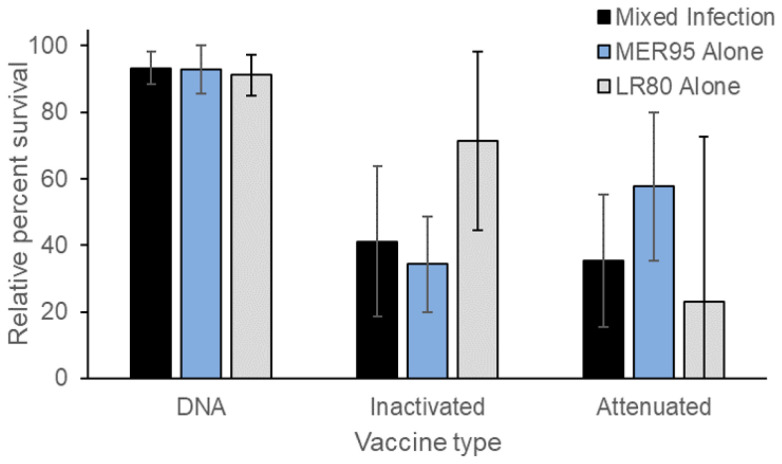
Relative percent survival (RPS) of vaccinated fish. Bars show mean relative percent survival (±1 standard error of mean) of vaccinated fish compared to unvaccinated fish for each vaccine regimen (x-axis), exposed to a mixed (black) or single infection of IHNV genotypes LR80 (gray) or MER95 (blue). The two genotypes were at a 1:1 ratio in mixed infections. Mean values were generated for triplicate tanks of each treatment and standard error propagated across the RPS calculation (100*(1-cumulative percent mortality vaccinated/cumulative percent mortality vaccinated)).

The kinetics of fish mortality through time caused by single infections of IHNV genotypes LR80 and MER95 were compared to those in co-infection, in the presence and absence of vaccination. In general, mortality began around day 5 and plateaued around day 15, in all treatments (Figure 1). No mortality was observed in any of the no virus exposure control groups (21 tanks and 420 fish total), with the exception of one fish on day 34 in the DNA unvaccinated treatment (data not shown). As such, the no virus exposure control treatment (mock virus exposure) was not considered in further analyses. Instead, the analysis focused on vaccine reductions in mortality in vaccinated compared to unvaccinated treatments, after virus exposure. Vaccination significantly lowered the hazard of mortality for all three vaccine regimen types, regardless of virus treatment (vaccine main effect; DNA: *Z_1,359_* = −5.64, *p* < 0.001; inactivated: *Z_1,358_* = −2.24, *p* = 0.025; attenuated: *Z_1,356_* = −2.50, *p* = 0.012; Table S2). Although not directly compared, the three vaccine regimens qualitatively differed in their ability to reduce disease caused by IHNV (Figure 1). The log-hazard of death due to vaccination changed by the greatest amount for the DNA vaccination regimen compared to the attenuated and inactivated vaccination regimens (log-hazard ratio of death vaccinated/unvaccinated ± SE; DNA vaccine: −2.6 ± 0.44 < inactivated vaccine: −0.73 ± 0.33 < attenuated vaccine: −0.65 ± 0.26; Table S2). This suggests the DNA vaccine regimen provided better disease protection than the other vaccine regimens, which was reflected in the relative percent survival (RPS) values for each vaccine (Figure 2). Furthermore, RPS values indicated that protection was more uniform across virus treatments for the DNA vaccine compared to the other vaccine types. In general, the hazard of fish mortality was not significantly different between the two viral genotypes or between single and mixed infections (*p* > 0.05, Table S2). The only exception was for the attenuated vaccine and unvaccinated regimens, where fish infected with LR80 alone had a lower hazard of mortality than mixed-infection fish, regardless of vaccination status (virus main effect; attenuated vaccine: *Z_1,356_* = −3.04, *p* = 0.002; Table S2). This was partly due to a slightly reduced level of cumulative mortality in the LR80 attenuated unvaccinated group (10% mortality) compared to the other unvaccinated controls (30–35% mortality), although this was not statistically assessed (Figure 1). In summary, all vaccine regimens increased fish survival after exposure to IHNV, with the DNA vaccine regimen being the most effective and attenuated being the least effective.

### 3.2. Number of Fish Shedding over Time

**Figure 3:**
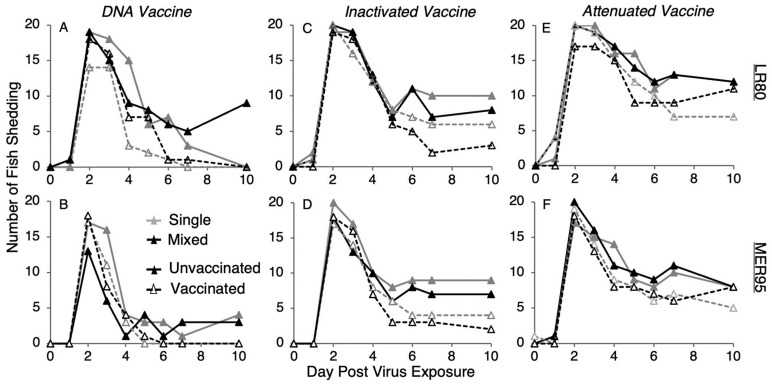
Effect of three vaccine regimens on the number of fish shedding IHNV. Lines represent raw data of the number of fish (out of 20) shedding IHNV genotypes through time (days) in single (gray triangles) or mixed infections (black triangles) in the presence of vaccination (dotted line and open symbols) or unvaccinated (solid line and filled symbols) treatments. Panels (**A**,**C**,**E**) show genotype and LR80, and panels (**B**,**D**,**F**) show genotype MER95 as measured by genotype-specific quantitative PCR. Vaccines used were DNA (**A**,**B**), Inactivated (**C**,**D**), and Attenuated (**E**,**F**). Symbols show sampling time points. Legend in panel (**B**) applies to all panels. Water samples continued to be taken from fish postmortem and were included in the figure.

**Figure 4:**
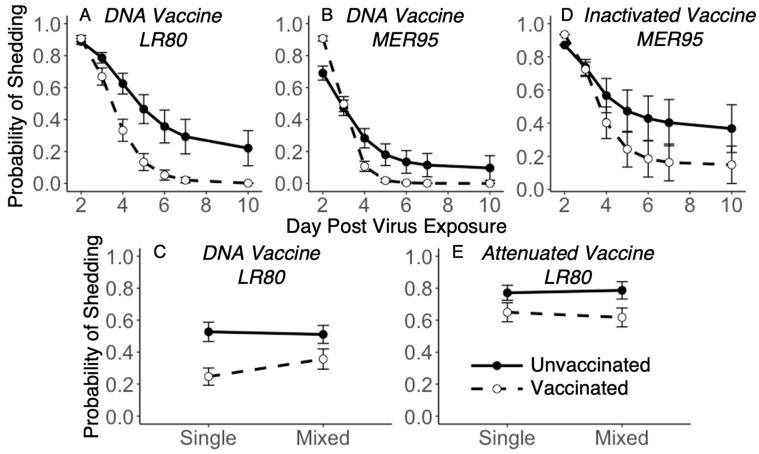
Probability of fish shedding interaction plots. Points show mean fitted probability of fish shedding IHNV per treatment ± 95% confidence interval (CI) from best-fit models for day*vaccine interaction (top panels (**A**,**B**,**D**)) and vaccine*infection type interaction (bottom panels (**C**,**E**)). In all panels, solid lines represent unvaccinated controls and dotted lines represent vaccinated treatments. For panels (**C**,**E**), the probabilities of shedding the target genotype in single (left) and mixed (right) infections are shown. Vaccine regimen and genotype of the model are given at the top of each panel (panels (**A**,**C**)—genotype LR80 in DNA vaccination treatments; panel (**B**)—genotype MER95 in DNA vaccination treatments; panel (**D**)—genotype MER95 in inactivated vaccine treatments; and panel (**E**)—genotype LR80 in attenuated vaccine treatments). Lines demonstrate trends but do not imply that data was collected between points. Points where CI bars do not overlap are considered significantly different.

To estimate the shedding prevention efficacy of the three vaccine regimen types, the number of fish shedding each of the two IHNV genotypes through time was quantified. Overall, the number of fish shedding IHNV peaked by day 2, and then decreased through time. By day 10, approximatively 50% of fish had stopped shedding, regardless of vaccine regimen or virus treatment (Figure 3). The analysis of the DNA vaccine regimen data revealed that there was a significant decrease in the probability of shedding from day 2 to 10, with the decrease being faster for vaccinated compared to unvaccinated fish for both virus genotypes (Figure 4A,B; day × vaccine interaction; LR80: *Z_1,551_* = −3.42, *p* < 0.001; MER95: *Z_1,552_* = −3.63, *p* < 0.001; Table S3A). For IHNV genotype MER95, the probability of fish shedding virus was initially higher for the DNA vaccinated compared to the unvaccinated treatment. By the end of the study period, the probability of shedding was higher in unvaccinated fish (Figure 4B). The probability of shedding genotype MER95 was also reduced by co-infection, regardless of DNA vaccination status (virus main effect; MER95: *Z_1,552_* = −0.82, *p* = 0.032; Table S3A). For genotype LR80, DNA vaccination reduced the overall probability of fish shedding, with a suggestive trend of the reduction being larger in single compared to mixed infections (Figure 4C; interaction virus × vaccine; LR80: *Z_1,551_* = 2.48, *p* = 0.013; Table S3A). This resulted in the probability of shedding LR80 being slightly higher in co-infection compared to single infection, but only after DNA vaccine treatment.

For the inactivated vaccine regimen, the analysis again revealed that, overall, there was a significant decrease in the probability of virus shedding through time from day 2 to 10 (Figure 3C,D; day main effect; LR80: *Z_1,554_* = −6.35, *p* < 0.001; Table S3B). For genotype MER95, the probability of shedding decreased faster through time for vaccinated compared to unvaccinated fish (Figure 4D; interaction day × vaccine; MER95: *Z_1,551_* = −2.53, *p* = 0.012; Table S3B). Similar to DNA vaccination, the initial probability of shedding was slightly higher for MER95 in vaccinated fish, but was lower than in unvaccinated fish by the end of the study period, although the difference was not significant (Figure 4D). There was not a significant vaccine effect for LR80 (*p* > 0.05, Table S3B), despite a suggestive trend of faster clearance with vaccination (Figure 3C). There was no indication that co-infection impacted the probability of shedding compared to single infections, for either genotype in the inactivated vaccine and unvaccinated treatments (Table S3B).

For the attenuated vaccine regimen, again in general, the probability of shedding decreased through time (Figure 3E,F; day main effect; LR80: *Z_1,552_* = −3.801, *p* < 0.001, MER95: *Z_1,548_* = −6.52, *p* < 0.001; Table S3C). Unlike the other vaccines, this decrease through time was not faster for vaccinated fish (*p* > 0.05; Table S3C). Vaccination did reduce the overall probability of fish shedding genotype LR80, with the reduction being slightly larger in mixed compared to single infection (Figure 4E; interaction virus × vaccine; LR80: *Z_1,552_* = 2.07, *p* = 0.039; Table S3C). There was no significant effect of attenuated vaccination or virus co-infection for genotype MER95 (*p* > 0.05, Table S3C).

Qualitatively, the DNA vaccine regimen provided the greatest reductions in the probability of shedding and the attenuated vaccine the least, similar to observed for disease protection. The DNA vaccine was the only regimen to reduce shedding to zero by the end of the study period. Whereas, 25% and 50% of fish were still shedding on day 10 for the inactivated and attenuated vaccine regimens, respectively (Figure 3).

### 3.3. Virus Shedding Intensity over Time

**Figure 5:**
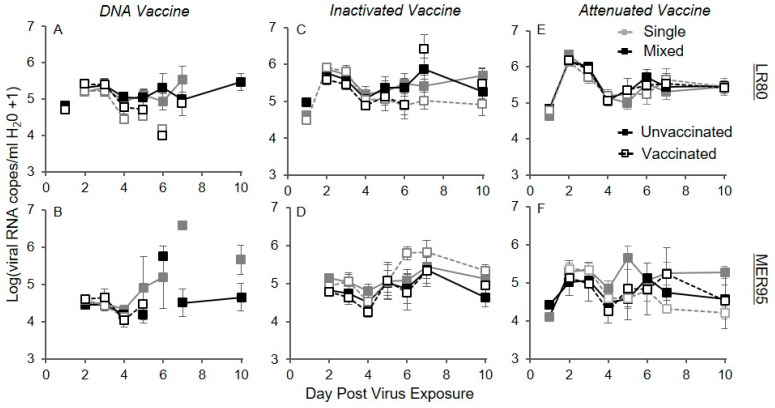
Effect of three vaccine regimens on shedding intensity of IHNV. Lines represent raw data of mean intensity (±1 S.E.) of shedding of IHNV (out of 20) genotypes through time (days) in single (gray squares) or mixed infections (black squares) in the presence of vaccination (dotted line and open symbols) or unvaccinated (solid line and filled symbols) treatments. Panels (**A**,**C**,**E**) show genotype and LR80, and panels (**B**,**D**,**F**) show genotype MER95 as measured by genotype-specific quantitative PCR. Vaccines used were DNA (**A**,**B**), inactivated (**C**,**D**), and attenuated (**E**,**F**). Only fish with detectable shedding are included in the mean, and markers are not connected to the line if fewer than 3 fish were shedding. The number of fish included in the mean can be found in Figure 2. The legend in panel (**E**) applies to all panels.

**Figure 6:**
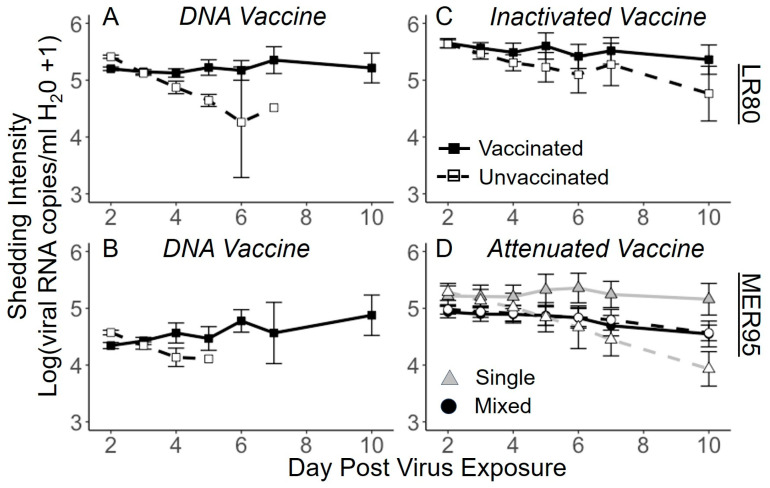
Shedding intensity interaction plots. Points show fitted mean treatment values of shedding intensity (log_10_(viral RNA copies/mL H_2_O +1) ± 95% confidence interval (CI) for interaction between day post-virus exposure (X-axis) and vaccine type (lines), from best-fit models. For all panels, unvaccinated fish are shown by a solid line and symbols, and vaccinated fish by a dashed line and open symbols. Top panels (**A**,**C**) show genotype LR80, and bottom panels (**B**,**D**) show genotype MER95. The vaccine regimen is given at the top of each panel. For panel (**D**), single infections are shown by gray triangles and mixed infections by black circles, due to the 3-way interaction between day*virus*vaccine. For all other panels, infection type was not significant in the model, and single and mixed infections are combined in the fitted mean values. Lines demonstrate trends but do not imply that data was collected between points. Where points are missing, no fish were shedding in the raw data, so predicted values for shedding intensity were not generated. Points where CI bars do not overlap are considered significantly different.

To assess the impact of vaccination on the amount of virus shed, shedding intensity (amount of virus shed from fish with detectable virus) over the course of infection was analyzed. In general, shedding intensity through time was more stable from days 2 to 10, compared to the probability of shedding (Figure 5). In other words, although fewer fish shed through time, those that did shed were shedding at similar rates. There were, however, some notable treatment effects on shedding intensity. For the DNA vaccine regimen, the analysis revealed that shedding intensity of both virus genotypes decreased through time in vaccinated fish but remained elevated in unvaccinated fish (Figure 6A,B; interaction day × vaccine; LR80: *t_1,150_* = −4.87, *p* < 0.001; MER95: *t_1,67_* = −4.02, *p* < 0.001, Table S4A). This resulted in shedding intensity being significantly lower in DNA vaccinated compared to unvaccinated fish by the end of the infection period for which shedding occurred, despite being initially slightly higher in vaccinated fish. There was no effect of co-infection on the intensity of shedding for either viral genotype (*p* > 0.05; Table S4A).

For the inactivated vaccine regimen, shedding intensity of genotype LR80 decreased through time in vaccinated fish but remained stable in unvaccinated fish (Figure 6C; interaction day × vaccine; LR80: *t_1,233_* = −2.92, *p* = 0.004, Table S4B). However, the intensity of MER95 did not change through time, regardless of vaccination status (*p* > 0.05, Table S4B). Co-infection reduced the intensity of shedding for genotype MER95 (virus main effect; MER95: *t_1,77_* = −2.17 *p* = 0.033) but not LR80 (*p* > 0.05, Table S4B).

For the attenuated vaccine regimen, the intensity of shedding decreased through time for LR80, but neither vaccination nor co-infection had any effect on the shedding intensity of the genotype (day main effect; LR80: *t_1,314_* = −9.59, *p* < 0.001, Table S4C). For genotype MER95, shedding intensity depended on co-infection and vaccine treatment (Figure 6D; day × virus × vaccine interaction; MER95: *t_1,224_* = −3.26, *p* = 0.001, Table S4C). Shedding intensity decreased most quickly through time for MER95 in the single-infection vaccine treatment group. The rate of decrease in shedding intensity was similar for the other attenuated vaccine regimen treatments and relatively small. The analysis also indicated that the initial shedding intensity was higher for MER95 in single compared to mixed infections, for both vaccinated and unvaccinated fish. Ultimately, by the end of the study period, for genotype MER95, shedding intensity was lowest in attenuated vaccinated single infections and highest in unvaccinated single infections, with co-infected fish having similar intermediate shedding intensity values.

Qualitatively, in contrast to mortality and shedding probability, the vaccine regimens had similar effects on shedding intensity. Reductions in shedding intensity were generally less than an order of magnitude on the log scale, hovering between 10^4^ and 10^6^ viral RNA copies/mL water shed over a 22-h period (Figure 5).

### 3.4. Cumulative Virus Shed

**Figure 7:**
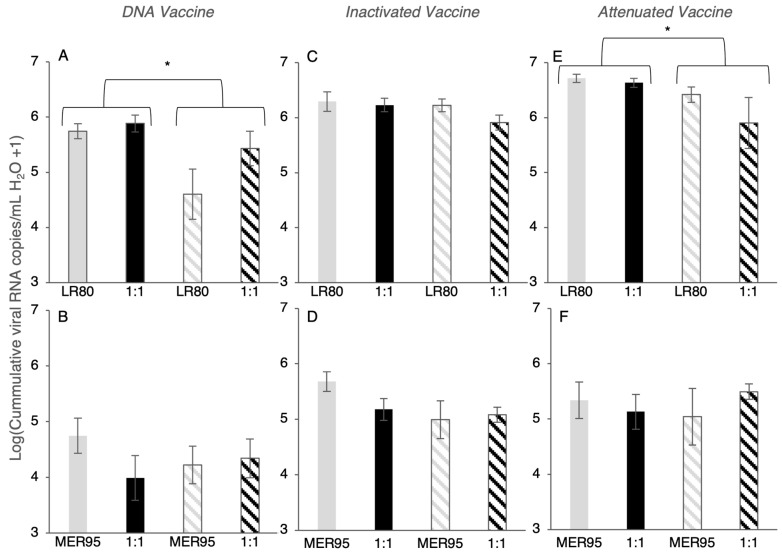
Cumulative amount of IHNV shed. Bars show total virus copies (Log_10_ (cumulative viral RNA copies +1)) summed across all days for each fish, then averaged across fish for IHNV genotypes LR80 (**A**,**C**,**E**) and MER95 (**B**,**D**,**F**), alone (gray bars) or in co-infection with the other genotype (black bars). Values are measured by genotype-specific quantitative PCR, and as such, mixed infections only contain the target genotype. Solid bars correspond to fish receiving the unvaccinated treatment, and striped bars show fish vaccinated against IHNV. Error bars show ±1 standard error (SE), and whiskers with (*) show statistical differences at *p* < 0.05 (see text).

The impact of the three vaccine types on cumulative virus shedding over 10 days was evaluated across all fish (Figure 7), which is akin to herd- or population-level shedding. Vaccination with the DNA and attenuated vaccine regimens significantly reduced total viral shedding for genotype LR80 (Figure 7A,E; vaccine main effect; LR80 DNA: *F_1,78_* = 7.16, *p* = 0.009; LR80 attenuated: *F_1,78_* = 4.31, *p* = 0.04; Table S5A,C), but not genotype MER95 (Figure 7B,F; null model best fit both vaccines, Table S5A,C). The inactivated vaccine regimen did not reduce cumulative virus shed for either IHNV genotype, despite a suggestive trend for MER95 (Figure 7C,D; LR80: null model best fit; MER95: *F_1,76_* = 2.6, *p* = 0.055, Table S5B). Total shedding was not significantly different between co-infection compared to single infection, for any viral genotype, regardless of vaccination status, despite suggestive trends for some treatments (Figure 7 and Table S5). In general, total shedding of MER95 was lower than LR80, but this was not analyzed directly (Figure 7). Qualitatively, the DNA vaccine reduced cumulative shedding over the course of the infection by the most, with reductions by the inactivated and attenuated vaccines being minimal.

### 3.5. Transmission Protection

**Figure 8:**
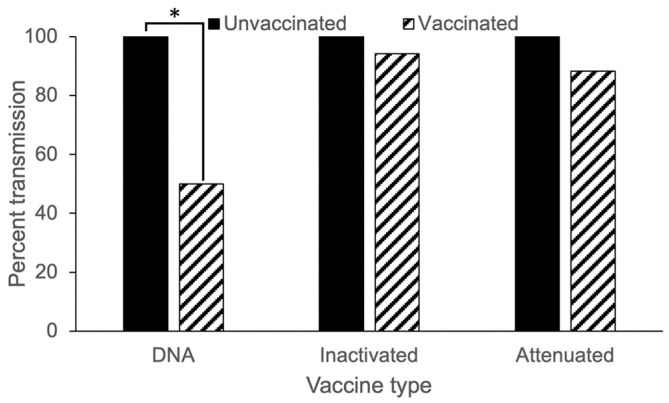
Transmission success in cohabitation assays. Bars show the percent of unvaccinated (solid) or vaccinated (striped) recipient tanks containing fish that became infected and shed IHNV after being placed in cohabitation with infected donor fish. N = 18 groups of 3 fish in all cases except attenuated and inactivated vaccine treatments, where N = 17. All donor fish tanks were positive for viral shedding (data not shown). Whiskers with (*) show statistical differences at *p* < 0.05 (see text).

**Figure 9:**
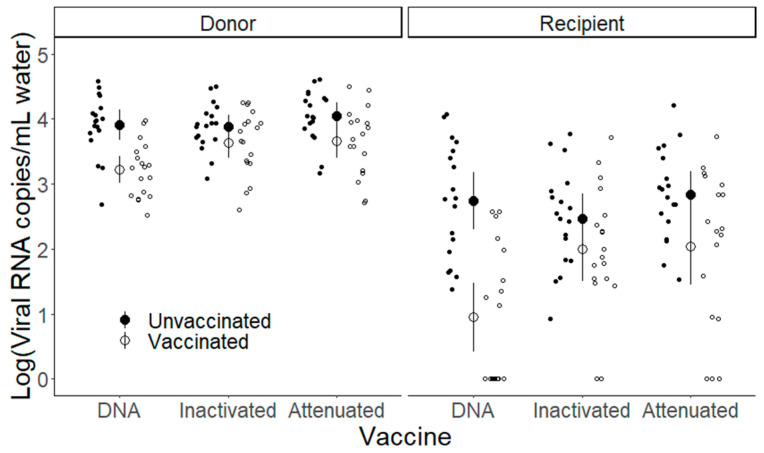
Viral shedding quantity of donor and recipient fish in cohabitation transmission assays. Small symbols show viral shedding quantity (Log_10_(viral RNA copies/mL H_2_O +1]) of individual tanks containing 3 unvaccinated (open symbols) or vaccinated (solid symbols) fish for each vaccine regimen (x-axis). The donor fish are shown in the left panel, and the recipient fish are shown in the right. Large symbols show the mean (±95% CI) for each treatment group.

In the cohabitation transmission assays, all replicate donor fish groups were found to be shedding virus regardless of vaccination regimen (Figure 8). When IHNV-infected donor fish were placed in direct cohabitation with uninfected recipient fish, under the same vaccine regimen, transmission success was 100% for all unvaccinated fish (Figure 8). When examining the number of recipient cohabitation replicates that became infected and shed virus, the DNA vaccine significantly reduced transmission by 50% (Figure 8, *χ^2^* = 9.48, *p* = 0.002). Neither the attenuated (Figure 8, *χ^2^* = 0.59, *p* = 0.44) nor the inactivated (Figure 8, *χ^2^* = 0.001, *p* = 0.98) vaccine regimens significantly reduced transmission. When examining the quantity of virus shed by cohabitation replicates (Figure 9), in almost all cases the recipient fish were found to shed significantly less virus than the donor fish (fish type main effect; inactivated vaccine: *t_1,34_* = −10.21, *p* < 0.001, attenuated vaccine: *t_1,34_* = −9.38, *p* < 0.001; Table S6). The exception was for the DNA vaccine regimen in which the vaccinated donor fish shed similar amounts to the unvaccinated recipient fish (vaccine status x fish type interaction: *t_1,34_* = −3.54, *p* = 0.001; Tables S6 and S7 pairwise comparisons). Vaccination resulted in significantly reduced shedding of both donor and recipient fish (compared to unvaccinated fish) for the attenuated vaccine (vaccine status main effect: *t_1,34_* = −2.73, *p* = 0.01; Table S6). This was also true for the DNA vaccinated fish, again with the exception of the vaccinated donor compared to unvaccinated recipient fish (vaccine status × fish type interaction: *t_1,34_* = −3.54, *p* = 0.001; Tables S6 and S7 pairwise comparisons). The inactivated vaccine did not result in significantly reduced viral shedding for either donor or recipient fish, compared to unvaccinated fish, despite a suggestive trend (vaccine status main effect: *t_1,34_* = −1.81, *p* = 0.08; Table S6). Qualitatively, the vaccine regimen (DNA) that resulted in the greatest reductions in the quantity of virus shed from donors also resulted in the largest reduction in transmission probability to recipient fish.

## 4. Discussion

All vaccine regimen types (DNA, inactivated, and attenuated) studied here significantly reduced clinical disease (mortality) caused by IHNV in rainbow trout. Efficacy qualitatively differed between the vaccine regimens, with the DNA vaccine consistently providing the greatest disease protection and the attenuated vaccine the least and most variable, although the vaccines were not directly statistically compared to each other. These findings are in agreement with previous studies, which found that the DNA vaccine most reliably eliminated mortality caused by IHNV in salmonids [18]. In the present study, the vaccines were much less effective at reducing viral shedding from fish compared to mortality, with the DNA vaccine again qualitatively being the most effective. While some notable effects of the vaccines on shedding were observed, usually in the form of faster viral clearance, these impacts were generally small. Overall, total shedding over the course of infection was only significantly reduced by two of the vaccine types against one of the viral genotypes. In cases where total shedding was reduced, it still ranged from 10^7^ to 10^9^ viral RNA copies over 10 days if the entire tank volume was considered. This was nearly equivalent to the amount of virus that unvaccinated fish shed. The transmission assay experiments confirmed that the three vaccine types provided minimal protection against IHNV transmission, with only the DNA vaccine resulting in a significant 50% decrease. The transmission reduction efficacy of the DNA vaccine appeared to be largely driven by a reduction in shedding intensity from donor fish by an order of magnitude. Collectively, these results provide additional support for IHNV being primarily transmitted horizontally through viral shedding into water [1,73,74,82], and thus, shedding quantification serves as a good proxy for transmission. Although these vaccines may provide beneficial protection against disease, they likely provide minimal immunity against infection and transmission in the field. These results are in line with our previous findings [47,56] and those of others [21,28].

A fundamental question is how IHNV vaccines can effectively reduce clinical disease but still allow viral shedding and transmission. Our results indicate that disease protection provided by vaccination was generally a function of more rapid viral clearance rather than infection blocking. In other words, the vaccines were non-sterilizing. This is based on our finding that the initial probability of shedding virus was similar between vaccinated and unvaccinated fish, but the shedding probability decreased more rapidly through time in vaccinated fish. Furthermore, although fewer fish were shedding as a result of vaccination, those fish actively shedding were generally reaching similar intensities. This translates to low reductions in transmission as exemplified by the cohabitation studies. We note that transmission was only assessed at one time point, at the peak of viral shedding. As such, the faster viral clearance, particularly of the DNA vaccine, might provide additional transmission reduction benefits, despite a lack of complete prevention. In general, as frequently observed in rainbow trout across a range of viral dosages, isolates, and host lineages, shedding of IHNV was highly acute and largely ended by day 5 post-infection [47,59,60,83]. Given that the majority of the virus is shed during the first three days post-infection [59,60], more rapid viral clearance provided by vaccination resulted in only small reductions in peak and total shedding. The transmission benefits of more rapid viral clearance, over the entire course of infection, are also likely to be small. Collectively, our results suggest that the vaccines induced a viral tolerance response in fish rather than a resistance response [84], in that fish experience similar infection levels but less disease, as we have observed previously [47]. Furthermore, our results support findings by ourselves and others that disease caused by IHNV is largely driven by how long fish maintain high levels of viremia, rather than peak viral loads [59,85].

There was some indication that vaccine efficacy depended on viral genotype. This was most evident when examining vaccine impacts on shedding. When examining total viral shedding, there was a general trend of the vaccines reducing isolate LR80 shedding more than MER95. The effect of viral genotype on the probability and intensity of shedding through time was less consistent. In fact, there was a suggestive trend that the DNA and inactivated vaccines initially increased the probability of MER95 shedding. However, this trend was only significant for one vaccine and time point; as such, we believe it was likely an artifact of fish-to-fish variation. Studies in other systems have indicated that vaccine efficacy is dependent on the virulence and genetic relatedness of vaccine and challenge strains [62,86]. In this study, the two viral genotypes caused similar levels of mortality in the absence of vaccination, ranging from 40 to 60%, and thus were similar in virulence. This is in line with what others have previously observed, with LR80 having slightly greater infectivity and virulence depending on host lineage [60,68]. Both viral genotypes are genetically heterologous to the vaccine strain WRAC, with MER95 being more dissimilar than LR80 [69]. We intentionally conducted heterologous challenges to reflect the high diversity of IHNV in the field and the likelihood of fish being exposed to genotypes different than those used for vaccination [50]. Antigenic differences between isolates of IHNV have been observed [87,88,89], and this could explain why vaccine efficacy was slightly higher against LR80. We note that the genetic relatedness between isolates was high (approximately 98.9%), indicating that antigenic differences may be low. However, antigen specificity was not directly measured here and will depend on the location of genetic mutations. Previous studies of IHNV have indicated that vaccine efficacy is stronger against homologous compared to heterologous challenge once initial innate immunity wanes and adaptive immunity develops, despite high cross-reaction of neutralizing antibodies [11,23,36,90,91,92,93]. However, protection efficacy in these previous studies was high even when viral isolates were more genetically distant from the vaccine strain than those tested here. It should be noted that these previous studies largely focused on disease rather than viral infection and shedding. Here, we also observed little evidence for differences in the level of clinical disease protection provided (survival) by vaccination against the two viral genotypes, despite shedding protection differences. It may be that these viral genotype effects are only apparent for viral transmission parameters [57,58], and this warrants further investigation.

We observed some trends suggesting mixed infections may impact vaccine efficacy, as has been observed in other systems [52,53,54]. Co-infection almost always caused the highest levels of disease, regardless of vaccine status, although the differences from single infections were only statistically significant with the attenuated vaccine regimen for genotype LR80. There was also some indication that vaccine efficacy against viral clearance was diminished in co-infection, for both IHNV genotypes. However, the collective impact of co-infection on total shedding was not significant, despite some suggestive trends. We have previously observed that in the absence of vaccination, co-infection impacts on viral fitness may depend on the relative virulence of genotypes [57,60,72,75,91,94,95]. Whether virulence is an important determinant for the impacts of co-infection on IHNV vaccine efficacy, as observed in other systems [62], is unknown and warrants further investigation. This could have implications for efficacy in the field where co-infection is common.

The maintenance of shedding and transmission in vaccinated fish could have major implications for viral ecology and evolution. Most immediately, the vaccination strategies evaluated here do not provide sterilizing immunity, which should be considered during fish transport and movement. Theory ascertains that more quickly replicating strains that can maximize shedding before immune clearance are likely to have the selective advantage [96]. If these rapidly replicating strains are also more virulent, as has been observed in many systems [57,58,60,63,97,98,99,100], this may select for increased pathogen virulence. Vaccination may amplify this effect by reducing the host mortality cost of virulence to the virus, particularly, as observed here, if there is potential for transmission from vaccinated hosts [61]. The transmission potential of vaccinated hosts may have been even more pronounced than we estimated here because shedding from dead fish was also included in the analysis. We included dead fish because previous studies indicate fish continue to shed virus after death [59,60], and virus can remain infectious for at least 3 days [75]. If dead fish are removed from the environment quickly, as typically occurs in aquaculture, their contribution to transmission may be reduced. This could make the relative reduction in shedding due to vaccination even smaller, because the vast majority of fish mortality occurred in the unvaccinated groups. We note that mortality in the shedding experiment was low, and typically occurred after day 5 when shedding had most ended, so the effect of mortality on shedding was likely minimal. It is unknown whether the IHNV vaccine drives virulence evolution, and this is a knowledge gap that requires investigation. Given the lack of sterilizing immunity, the gains of fish survival should be weighed against the potential risk of emergence of more virulent IHNV strains [100,101,102].

In general, there is little published information available on the field usage and delivery routes of IHNV vaccines. The most well-documented case is a commercial version of the DNA vaccine, APEX-IHN^®^ (Intervet Canada Corp., Kirkland, QC, Canada), which has been primarily delivered intramuscularly in the Canadian Atlantic salmon aquaculture industry, due to demonstrated diminished efficacy via other delivery routes [10,34]. To our knowledge, there has been limited usage of inactivated vaccines in the field, but studies indicate that intraperitoneal delivery has the highest efficacy [14,20]. Field usage of the attenuated vaccine also remains undocumented, but of all vaccine types, it shows the most promise for immersion delivery. Although attenuated vaccines are typically more effective when delivered via injection compared to immersion [26], they often have the highest bath immersion efficacy of the vaccine types, making the immersion delivery route one of their primary advantages [18,27]. Furthermore, autogenous attenuated vaccines with reduced regulatory and reporting restrictions can be generated using standard cell culture techniques from viral strains circulating in aquaculture facilities, often making them more accessible to the industry [23,24,25,26]. Our discussions with the industry indicate that immersion of attenuated vaccines has been the most widely explored delivery route in the field. In contrast, the DNA and inactivated vaccines have been most commonly delivered by intramuscular and intraperitoneal injection, respectively. We used these delivery routes here to mostly closely reflect previous usage and the most promising trends in the field. We acknowledge that the use of different delivery methods may influence efficacy, as shown by others [19,26]. For this reason, throughout the study, statistical comparisons were only made between treatments within a given vaccine type (i.e., DNA vaccinated vs. unvaccinated). We note that regardless of our major findings holds that the vaccines, regimens still allowed for high levels of viral shedding and transmission, even under the most effective DNA vaccine injection delivery. The impacts of vaccine delivery route of transmission protection efficacy for each of the vaccine types warrants further investigations, but based on extensive previous studies comparing these and similar vaccines for protection against mortality [19], we do not expect efficacy to be greater than that observed for the DNA vaccine.

In addition to the delivery route, vaccine dosage can influence efficacy [11]. IHNV vaccine delivery dosage in the field is highly variable and not well documented. Indeed, for the only commercially available IHNV vaccine, Apex-IHNV, the dosage (DNA copies or equivalent) is proprietary. Here, we sought to use dosages comparable to previous published work in this system and biologically relevant to field usage [11,14,21,23,33]. We note that the disease protection efficacy (fish survival) was similar to what others have observed with these vaccines. Indeed, the DNA vaccine has been shown to provide strong levels of clinical disease protection at even lower dosages than used here [11]. The impact of vaccine dosage on protection against IHNV transmission warrants further investigation and may be an avenue to enhance transmission protection. Typically, vaccine efficacy is evaluated primarily based on clinical disease protection [19]. Our work highlights that vaccines delivered as dosages that provide strong clinical disease protection may still allow for viral transmission. As such, including assessment of transmission protection is likely to bolster inference of field efficacy and enhance prospects for viral management and eradication.

## 5. Conclusions

In conclusion, our results indicated that all three IHNV regimens tested here (DNA, inactivated, and attenuated) provided minimal protection against viral shedding, despite high clinical disease protection. Which IHNV vaccination strategy will provide the most effective long-term management in the field remains an open question. Answering this ultimately involves consideration of a variety of environmental, economic, regulatory, and political factors [103]. Our results indicate that the DNA vaccine regimen most effectively manages IHN disease, viral shedding, and transmission in laboratory trials. By shortening the infectious period, the vaccine might be able to reduce the size and duration of epidemics [104] and will almost certainly alleviate the level of IHN disease and mortality in vaccinated fish. However, even DNA vaccination may be unable to entirely prevent epidemics without fully blocking viral transmission, and this could result in selective pressure on vaccine resistance [64] and virulence evolution [61]. Whether this will outweigh the benefits of vaccination is uncertain [105]. Others have experienced success with IHNV DNA vaccination in the field [38], which appears to effectively reduce transmission in Atlantic salmon (*Salmo salar*) [33]. However, vaccine transmission-blocking efficacy may depend on host species [48], particularly given that Atlantic salmon are an exotic host for the virus [49,106]. Many vaccine types have been explored for their usage in the field, with autogenous attenuated IHNV vaccines showing particularly high interest due to regulatory as well as economic advantages, despite often lower disease protection [18]. Our results suggest these attenuated vaccines also likely provide lower transmission protection and may be even less effective for pathogen eradiation [107]. Our work does indicate that injection vaccines are most effective against IHNV, as observed by others [10,18,26,28]. However, even with these vaccines, high levels of viral shedding and substantial transmission were maintained, and as such, development of effective transmission-blocking vaccines, which allow for large-scale delivery, continues to be an important priority [18,27]. Assessing transmission prevention efficacy of vaccines is a first step to achieving this goal, with implementation of strategies such as altering vaccine dosages or administering adjuvants potentially providing additional benefits [21]. Vaccines are a highly effective method for managing a variety of pathogens, and improving their transmission protection efficacy is likely to enhance these benefits, particularly in cases where they have performed sub-optimally in the field.

## Data Availability

All data not published in the manuscript or supplement is available from the corresponding author upon request.

## Supplementary Materials

The following supporting information can be downloaded at https://www.mdpi.com/article/10.3390/vaccines13080864/s1, Table S1. Summary of experimental design and the number of replicates per treatment. Table S2. Parameter estimates of the best fit models from fish survival (Coxme) analyses. Table S3. Parameter estimates (relative to baseline) from the best fit models for the number of fish shedding (2–10 days post-exposure, GLMER) analyses. Table S4. Parameter estimates for the best fit models of the intensity of IHNV shedding over time (2–10 days post-exposure, lme). Table S5. Parameter estimates for the best fit models of the cumulative amount of IHNV shed analyses (2–10 days post-exposure, ANOVA). Table S6. Parameter estimates for the best fit models of the cohabitation transmission assay shedding amount analyses (lme). Table S7. Tukey’s pairwise comparisons for DNA vaccine cohabitation transmission shedding analysis (values obtained from ‘emmeans’ package in R).
