# Supplementary material for: Efficacy of Three Vaccine Regimens Against Infectious Hematopoietic Necrosis Virus Transmission Potential in Rainbow Trout
Source: Vaccines (Basel). 2025 Aug 15;13(8):864. doi: 10.3390/vaccines13080864 (PMC12390467; doi:10.3390/vaccines13080864)
Supplement: Supplementary file 1 [file vaccines-13-00864-s001.zip › vaccines-3681205-supplementary.pdf]

Supplemental Materials

Table S1. Summary of experimental design and the number of replicates per treatment. Shedding mortalities column provides day and number of fish that died during the shedding experiment. Mortalities for the virulence challenge can be found in figure 1.

Vaccine treatment	Virus treatment	Infection type	# Fish per treatment		Shedding Mortalities
			Virulence challenge	Shedding	Day (# of fish)
DNA	LR80	Single	3 × 20	20	None
DNA	MER95	Single	3 × 20	20	None
DNA	1:1	Mixed	3 × 20	20	None
DNA	MEM10	Control	3 × 20	5	None
Sham DNA	LR80	Single	3 × 20	20	6 (1), 8(1)
Sham DNA	MER95	Single	3 × 20	20	4 (1), 7 (1), 8 (2)
Sham DNA	1:1	Mixed	3 × 20	20	5 (1), 6 (1), 8 (1), 10 (1)
Sham DNA	MEM10	Control	3 × 20	5	None
Inactivated	LR80	Single	3 × 20	20	5 (1), 6 (1), 8 (2)
Inactivated	MER95	Single	3 × 20	20	5 (2), 6 (1), 7 (1)
Inactivated	1:1	Mixed	3 × 20	20	5 (2)
Inactivated	MEM10	Control	3 × 20	5	None
Sham inactivated	LR80	Single	3 × 20	20	5 (1), 6 (3), 8 (3), 9 (3)
Sham inactivated	MER95	Single	3 × 20	20	5 (2), 6 (3), 7 (1), 8 (2), 10 (1)
Sham inactivated	1:1	Mixed	3 × 20	20	3 (1), 4 (1), 5 (3), 7 (2), 8 (1)
Sham inactivated	MEM10	Control	3 × 20	5	None
Attenuated	LR80	Single	3 × 20	20	5 (2), 7 (2), 10 (2)
Attenuated	MER95	Single	3 × 20	20	5 (1), 6 (1), 9 (1)
Attenuated	1:1	Mixed	3 × 20	20	5 (4),7 (2), 8 (1), 9 (1)
Attenuated	MEM10	Control	3 × 20	5	None
Sham attenuated	LR80	Single	3 × 20	20	5 (2), 7 (4), 8 (1), 9 (2), 10 (1)
Sham attenuated	MER95	Single	3 × 20	20	5 (6), 8 (1), 9 (2)
Sham attenuated	1:1	Mixed	3 × 20	20	4 (1), 5 (4), 6 (1), 7 (2), 8 (2), 9 (1)

Sham attenuated	MEM10	Control	3 × 20	5	None

Table S2. Parameter estimates of the best fit models from fish survival (Coxme) analyses. Each vaccine type, DNA (A), Inactivated (B), and Attenuated (C), was analyzed separately. Model is given with (*) symbol at bottom each panel, with AIC value. If another model that converged was within 2 AIC, it is also provided. If interactions are shown, main effects of terms were also included in model but are not shown in text. Response variable is only shown for best fit model, but same for all models. Note that the β term provides the log(hazard ratio) and that exp(β) provides hazard ratio values.

(A)	**Parameter**	**β**	**exp(β)**	**se(β)**	**z**	***P***
DNA vaccines	Vaccine	-2.60	0.07	0.44	-5.85	**< 0.001**
Number of events: 70 out of 359 fish						

*Model: coxme (day of death, censor) ~ Vaccine + (1|Tank); AIC: 738.48
-Alt model 1: ~ Virus + Vaccine + (1|Tank); AIC: 740.03

(B)	**Parameter**	**β**	**exp(β)**	**se(β)**	**z**	***P***
Inactivated vaccines	Vaccine	-0.73	0.48	0.33	-2.24	**0.025**
	Virus: LR80	-0.53	0.59	0.41	-1.30	0.20
	Virus: Mer95	0.03	1.03	0.38	0.07	0.94
Number of events: 87 out of 358 fish						

*Model: coxme (day of death, censor) ~ Virus + Vaccine + (1|Tank); AIC: 979.46
-Alt model 1: ~ Virus*Vaccine + (1|Tank); AIC: 981.20
-Alt model 2: ~ Virus + (1|Tank); AIC: 979.54
-Alt model 3: ~ Vaccine + (1|Tank); AIC: 979.70
Because two simplest models tied, the model with lowest AIC that included both terms was chosen. This did not change the statistical results.

(C)	**Parameter**	**β**	**exp(β)**	**se(β)**	**z**	***P***
Attenuated vaccines	Vaccine	-0.65	0.52	0.26	-2.50	**0.012**

Virus:LR80	-1.00	0.37	0.33	-3.04	0.002
Virus: Mer95	-0.45	0.64	0.29	-1.56	0.12

Number of events: 69 out of 356 fish

*Model: coxme (day of death, censor) ~ Virus + Vaccine + (1|Tank); AIC: 784.38
-Alt model 1: ~ Virus + (1|Tank); AIC: 786.35
-Alt model 2: ~ Vaccine + (1|Tank); AIC: 786.10
Because two simplest models tied, the model with lowest AIC that included both terms was chosen. This did not change the statistical results.

Table S3. Parameter estimates (relative to baseline) from the best fit models for the number of fish shedding (2-10 days post-exposure, GLMER) analyses. Baseline is set to vaccine = SHAM, virus = single infection of virus of focus. Each vaccine type, DNA (A), Inactivated (B), and Attenuated (C), was analyzed separately for each virus type, LR80 (top), MER95 (bottom). Model deviance and residual degrees of freedom are provided at the bottom of each table. Model structure is given with (*) symbol at bottom each panel, with AIC value. If another model that converged was within 2 AIC, it is also provided. If interactions are shown, main effects of terms were also included in model but are not shown in text.

(A) *DNA vaccines*	**Factor**	**Estimate**	**z**	***P***
**LR80**	Day	-0.84	-4.67	< 0.001
	Virus	-0.81	-1.46	0.14
	Vaccine	1.06	1.05	0.30
	Day × Vaccine	-0.90	-3.42	**< 0.001**
	Virus × Vaccine	1.97	2.48	**0.013**
Deviance: 461.3; Residual degrees of freedom: 551				
**MER95**	Day	-1.11	-4.76	< 0.0001
	Virus	-0.82	-2.14	**0.032**
	Vaccine	4.21	3.50	< 0.001
	Day × Vaccine	-1.36	-3.63	**< 0.001**
Deviance: 374.1; Residual degrees of freedom: 552				

*LR80 model: glmer (# pos., # neg.) ~ Day*Vaccine + Virus*Vaccine + (Day|Fish); AIC: 479.3

-LR80 Alt model: ~ Day*Vaccine + Virus*Vaccine + Day*Virus + (Day|Fish); AIC: 480.3
*MER95 model: glmer (# pos., # neg.) ~ Virus + Day*Vaccine + (Day|Fish); AIC: 390.11
-MER95 Alt model 1: ~ Day*Vaccine + Virus*Vaccine + (Day|Fish); AIC: 388.99
-MER95 Alt model 2: ~ Day*Vaccine + Virus*Vaccine + Day *Virus + (Day|Fish); AIC: 390.61

(B) *Inactivated vaccines*	**Factor**	**Estimate**	**z**	***P***
**LR80**	Day	-0.83	-6.354	< **0.001**
	Vaccine	-0.22	-0.61	0.54
Deviance: 515.3; Residual degrees of freedom: 554				
**MER95**	Day	-0.71	-2.88	0.004
	Vaccine	2.64	2.39	0.017
	Day × Vaccine	-0.95	-2.515	**0.012**
Deviance: 426.4; Residual degrees of freedom: 541				

*LR80 model: glmer (# pos., # neg.) ~ Day + Vaccine + (Day|Fish), AIC: 527.28
-LR80 Alt model 1: ~ Day + Virus + (Day|Fish); AIC: 527.34
-LR80 Alt model 2: ~ Day*Vaccine + (Day|Fish); AIC: 525.91
-LR80 Alt model 3: ~ Day* Virus + (Day|Fish); AIC: 526.82
-LR80 Alt model 4: ~ Virus + Day*Vaccine + (Day|Fish); AIC: 527.67
*MER95 model: glmer (# pos., # neg.) ~ Day*Vaccine + (Day|Fish); AIC: 440.36
-MER95 Alt model 1: ~ Virus + Day*Vaccine + (Day|Fish); AIC: 441.74

(C) *Attenuated vaccines*	**Factor**	**Estimate**	**z**	***P***
**LR80**	Day	-0.44	-3.801	<**0.001**
	Virus	-0.92	-1.037	0.30
	Vaccine	-2.29	-2.79	0.005
	Virus × Vaccine	-2.42	2.07	**0.039**
Deviance: 462.8; Residual degrees of freedom: 552				
**MER95**	Day	-1.74	-6.52	< **0.001**
Deviance: 482.7; Residual degrees of freedom: 548				

*LR80 model: glmer (# pos., # neg.) ~ Day + Virus*Vaccine + (Day|Fish), AIC: 478.83
-LR80 Alt model 1: ~ Day*Virus + Day*Vaccine + (Day|Fish); AIC: 478.75
-LR80 Alt model 2: ~ Day*Vaccine + Virus*Vaccine + Day*Virus*Vaccine + (Day|Fish); AIC: 479.88
-LR80 Alt model 3: ~ Day*Virus+ Virus*Vaccine + Day*Virus*Vaccine + (Day|Fish); AIC: 479.89
*MER95 model: glmer (# pos., # neg.) ~ Day + (Day|Fish); AIC: 492.74
-MER95 Alt model 1: ~ Day + Vaccine + (Day|Fish); AIC: 492.36

-MER95 Alt model 2: ~ Day + Virus + Vaccine + (Day|Fish); AIC: 494.15
-MER95 Alt model 3: ~ Day*Vaccine + (Day|Fish); AIC: 493.60

Table S4. Parameter estimates for the best fit models of the intensity of IHNV shedding over time (2-10 days post-exposure, lme). Each vaccine type, DNA (A), Inactivated (B), and Attenuated (C), was analyzed separately for each virus type, LR80 (top), MER95 (bottom).

(A) *DNA vaccines*	**Factor**	**Estimate**	**t-value**	***Residual Degrees of freedom***	***P***
**LR80**	Day	-0.03	-1.43	150	0.16
	Virus	0.14	1.65	73	0.10
	Vaccine	0.71	3.84	73	<0.001
	Day × Vaccine	-0.25	-4.87	150	**< 0.001**
**MER95**	Day	0.08	1.56	67	0.12
	Virus	0.20	1.01	69	0.31
	Vaccine	0.82	3.47	69	< 0.001
	Day × Virus	-0.08	-1.27	67	0.21
	Day × Vaccine	-0.30	-4.02	67	**< 0.001**

*LR80 model: lme (log(viral load) ~ Virus + Day*Vaccine + (Day|Fish) + weights=varPower, AIC: 382.91
-LR80 Alt model 1: ~ Virus* Vaccine + Day*Vaccine + (Day|Fish) + weights=varPower; AIC: 384.82
*MER95 model: lme (log(viral load) ~ Day*Virus + Day*Vaccine + (1|Fish) + weights=varPower, AIC: 225.85
-MER95 Alt model 1: ~ Day*Virus*Vaccine + (1|Fish) + weights=varPower, AIC: 226.74
-MER95 Alt model 2: ~ Virus*Vaccine + Day*Vaccine + Day*Virus + (1|Fish) + weights=varPower, AIC: 226.98

(B) *Inactivated vaccines*	**Factor**	**Estimate**	**t-value**	***Residual Degrees of freedom***	***P***
**LR80**	Day	-0.09	-4.02	233	< 0.001
	Vaccine	0.19	1.33	78	0.19
	Day × Vaccine	-0.10	-2.92	233	**0.004**

**MER95**	Virus	-0.27	-2.17	77	**0.033**

*LR80 model: lme (log(viral load) ~ Day*Vaccine + (Day|Fish) + weights=varPower, AIC: 609.60
-LR80 Alt model 1: ~ Virus + Day*Vaccine + (Day|Fish) + weights=varPower; AIC: 609.37
-LR80 Alt model 2: ~ Virus*Vaccine + Day*Vaccine + (Day|Fish) + weights=varPower; AIC: 609.98
-LR80 Alt model 3: ~ Day*Virus + Day*Vaccine + (Day|Fish) + weights=varPower; AIC: 611.31
*MER95 model: lme (log(viral load) ~ Virus + (1|Fish), AIC: 521.05
-MER95 Alt model 1: ~ Day + Virus + (Day|Fish) + weights=varPower, AIC: 521.95
-MER95 Alt model 2: ~ Virus + (1|Fish) + weights=varPower, AIC: 521.96
-MER95 Alt model 3: ~ Day + Virus + (1|Fish), AIC: 522.21
-MER95 Alt model 4: ~ Virus + Vaccine + (1|Fish), AIC: 522.54
-MER95 Alt model 4: ~ Day + Virus + (1|Fish) + weights=varPower, AIC: 522.91

(C) *Attenuated vaccines*	**Factor**	**Estimate**	**t-value**	***Residual Degrees of freedom***	***P***
**LR80**	Day	-0.16	-9.59	314	< **0.001**
**MER95**	Day	-0.06	-2.44	224	0.02
	Vaccine	0.03	0.15	71	0.89
	Virus	0.22	0.98	71	0.33
	Day × Virus	0.032	0.83	224	0.41
	Virus × Vaccine	0.41	1.26	71	0.21
	Day × Vaccine	0.001	0.025	224	0.98
	Day × Virus × Vaccine	-0.19	-3.26	224	**0.001**

*LR80 model: lme (log(viral load) ~ Day + (Day|Fish) + weights=varPower, AIC: 781.19
*MER95 model: lme (log(viral load) ~ Day*Virus*Vaccine + (1|Fish), AIC: 589.08
-MER95 Alt model 1: ~ Day*Virus*Vaccine + (1|Fish) + weights=varPower, AIC: 591.07

Table S5. Parameter estimates for the best fit models of the cumulative amount of IHNV shed analyses (2-10 days post-exposure, ANOVA). Each vaccine type, DNA (A), Inactivated (B), and Attenuated (C), was analyzed separately for each virus type, LR80 (top), MER95 (bottom).

(A) *DNA vaccines*	**Factor**	**df**	***F***	***P***
**LR80**	Vaccine	1	7.16	**0.009**
Residual degrees of freedom = 78				

**MER95**	Null	NA	NA	NA
Residual degrees of freedom = 79				

*LR80 model: aov (log(viral load) ~ Vaccine, AIC: 277.07
-LR80 Alt model 1: ~ Virus + Vaccine, AIC: 276.31
-LR80 Alt model 2: ~ Virus*Vaccine, AIC: 276.87
*MER95 model: aov (log(viral load) ~ 1, AIC: 301.74
-MER95 Alt model 1: ~ Virus, AIC: 302.89
-MER95 Alt model 2: ~ Vaccine, AIC: 303.68

(B) *Inactivated vaccines*	**Factor**	**df**	***F***	***P***
**LR80**	Null	NA	NA	NA
Residual degrees of freedom = 79				
**MER95**	Virus	1	2.60	0.055
Residual degrees of freedom = 76				

*LR80 model: aov (log(viral load) ~1, AIC: 156.64
-LR80 Alt model 1: ~ Vaccine, AIC: 156.73
-LR80 Alt model 2: ~ Virus, AIC: 156.81
-LR80 Alt model 3: ~ Virus + Vaccine, AIC: 156.85
-LR80 Alt model 4: ~ Virus*Vaccine, AIC: 157.99
*MER95 model: aov (log(viral load) ~ Virus, AIC: 195.53
-MER95 Alt model 1: ~ Vaccine, AIC: 197.13
-MER95 Alt model 2: ~ Virus + Vaccine, AIC: 195.21
-MER95 Alt model 3: ~ Virus*Vaccine, AIC: 196.11

(C) *Attenuated vaccines*	**Factor**	**df**	***F***	***P***
**LR80**	Vaccine	1	4.31	**0.04**
Residual degrees of freedom = 78				
**MER95**	Null	NA	NA	NA
Residual degrees of freedom = 78				

*LR80 model: aov (log(viral load) ~ Vaccine, AIC: 247.95
-LR80 Alt model 1: ~ Virus + Vaccine, AIC: 248.45
-LR80 Alt model 2: ~ Virus*Vaccine, AIC: 249.64
*MER95 model: aov (log(viral load) ~ 1, AIC: 283.66
-MER95 Alt model 1: ~ Virus, AIC: 285.65
-MER95 Alt model 2: ~ Vaccine, AIC: 285.38

Table S6. Parameter estimates for the best fit models of the cohabitation transmission assay shedding amount analyses (lme). Each vaccine type, DNA (A), Inactivated (B), and Attenuated (C), was analyzed separately. Vaccine status = unvaccinated (baseline) or vaccinated. Fish type = donor (baseline) or recipient.

(A) *DNA vaccines*	**Factor**	**Estimate**	**SE**	**t-value**	***Residual Degrees of freedom***	***P***
	Intercept	3.91	0.18	21.89	34	**<0.001**
	Vaccine status	-0.69	0.25	-2.73	34	**0.01**
	Fish type	-1.16	0.22	-5.28	34	**<0.001**
	Vaccine Status x Fish type	-1.11	0.31	-3.54	34	**0.0012**

*Model: lme(log(viral load+1) ~ Vaccine status*Fish type, random = 1|Tank, AIC: 170.12
-No alternative model within ΔAIC = 9.

(B) *Inactivated vaccines*	**Factor**	**Estimate**	**SE**	**t-value**	***Residual Degrees of freedom***	***P***
	Intercept	3.91	0.14	28.79	34	**<0.001**
	Vaccine status	-0.30	0.16	-1.81	34	0.08
	Fish type	-1.46	0.14	-10.28	34	**<0.001**

*Model: lme(log(viral load+1) ~ Vaccine status + Fish type, random = 1|Tank, AIC: 146.5
-Alt model 1: lme(log(viral load+1) ~ Fish type, random = 1|Tank, AIC: 147.8.
-Alt model 2: lme(log(viral load+1) ~ Vaccine status*Fish type, random = 1|Tank, AIC: 148.3.

(C) *Attenuated vaccines*	**Factor**	**Estimate**	**SE**	**t-value**	***Residual Degrees of freedom***	***P***
	Intercept	4.12	0.16	26.30	34	**<0.001**
	Vaccine status	-0.54	0.20	-2.73	34	**0.01**
	Fish type	-1.36	0.15	-9.38	34	**<0.001**

*Model: lme(log(viral load+1) ~ Vaccine status + Fish type, random = 1|Tank, AIC: 160.4
-Alt model 1: lme(log(viral load+1) ~ Vaccine status*Fish type, random = 1|Tank, AIC: 161.2.

Table S7. Tukey's pairwise comparisons for DNA vaccine cohabitation transmission shedding analysis (values obtained from 'emmeans' package in R).

DNA vaccines **Contrast**	**Estimate**	**SE**	**t-ratio**	***Residual Degrees of freedom***	***P***
Unvaccinated donor – Vaccinated donor	0.69	0.25	2.73	34	**0.047**
Unvaccinated donor – Unvaccinated recipient	1.16	0.22	5.28	34	**<0.001**
Unvaccinated donor – Vaccinated recipient	2.96	0.25	11.73	34	**<0.001**
Vaccinated donor – Unvaccinated recipient	0.48	0.25	1.88	34	0.254
Vaccinated donor – Vaccinated recipient	2.27	0.22	10.29	34	**<0.001**
Unvaccinated recipient – Vaccinated recipient	1.80	0.25	7.11	34	**<0.001**
